# Supplementary material for: Quantitative Analytical and Computational Workflow for Large-Scale Targeted Plasma Metabolomics
Source: Metabolites. 2023 Jul 13;13(7):844. doi: 10.3390/metabo13070844 (PMC10383057; doi:10.3390/metabo13070844)
Supplement: Supplementary file 1 [file metabolites-13-00844-s001.zip › Figure S1.pdf]

Supplementary Figures

Quantitative analytical and computational workflow for large-scale targeted plasma metabolomics

*Antonia Fecke, Nay Min Min Thaw Saw, Dipali Kale, Siva Swapna Kasarla, Albert Sickmann, Prasad Phapale**

Supplementary Figure 1

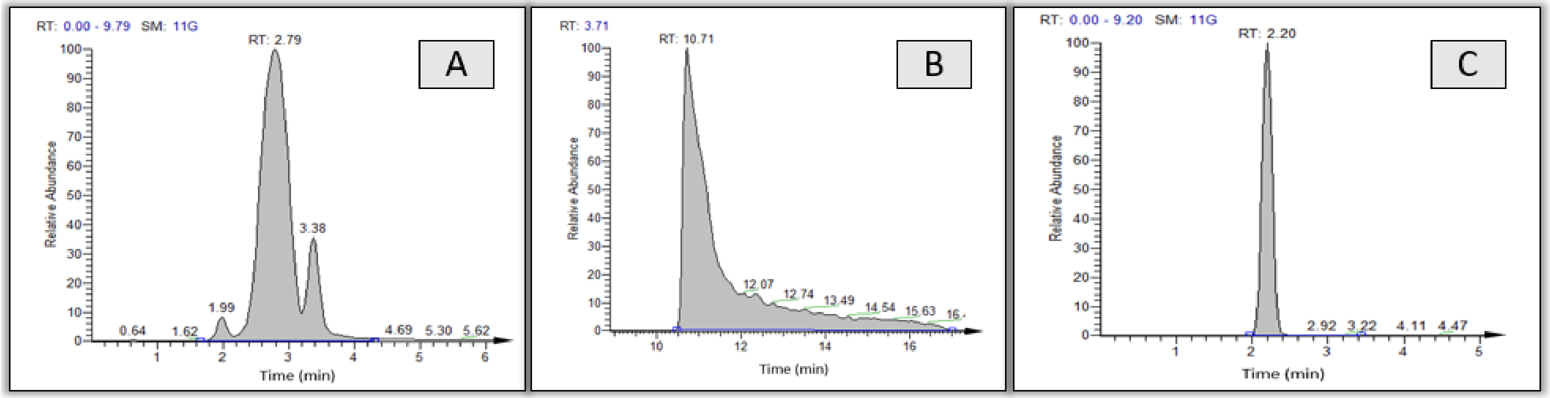

Supplementary Figure 1: Example of the categories assigned during manual curation of peak shapes. A shows an example of a split peak which is split close to the baseline, B and example of a broad peak and C and example of a good peak.
